# The Shortage of Doctors

**Published:** 1918-01-12

**Authors:** Gerald H. Fitzgerald

**Affiliations:** Guy's Hospital, S.E. 1.


### THE EDITOR'S LETTER-BOX.

### The Shortage of Doctors.

*To the Editor of* The Hospital.

Sir,—The lay press is once again growing eloquent upon the effects likely to be produced by a shortage of doctors. Upon this occasion a certain weight has been lent to its gloomy prognostications by the announcement of Sir Donald MacAlister at the General Medical Council to the effect that instead of 1,100 men preparing to qualify in 1919, there are in prospect only 572 men and 261 women, a shortage of over 25 per cent. But before a shortage can be assumed two facts must be ascertained: Firstly, was the pre-war rate of production one accurately and exactly adjusted to pre-war needs? and secondly, is there no other source of supply but from English and Scotch training-schools? With regard to the first point, so far from being accurately adjusted, the supply in pre-war days largely exceeded the demand, as can be shown by the fact that in spite of the drain of young medical men into the R.A.M.C. private practices and institutions are carrying on quite comfortably, if at a slightly higher pressure, the demand for medical men in institutions, as evidenced by advertisements in the columns of the *British Medical Journal* and the *Lancet* is no greater than in a corresponding period of 1913, nor are more assistants required in private practices than in that year.

Secondly, there is in London at the present time a considerable number of medical men, largely Irish practitioners attracted to this country by newspaper stories of shortage and high *locum* fees, who are unable to find work of any kind. A visit to the principal transfer and *locum* agents will convince anyone of this fact. There exists, moreover, in Ireland a considerable surplus of qualified men, amply sufficient to fill any posts which may become vacant owing to war necessities for some time to come. Further, as increasing numbers of doctors in the R.A.M.C. leave the Service owing to sickness and other causes, the supply of available men will automatically increase, until, in the inevitable disorganisation that will follow the conclusion of hostilities, far from there being any shortage, there will be an enormous surplus of men, eager for work upon any terms. The access of increasing numbers of women to the profession, who, notwithstanding the activities of the B.M.A., can generally be relied upon to work for less than their male colleagues, cannot but have a prejudicial effect upon the financial situation. The incidental costs of medical education are naturally more than doubled by the war; the outlook in medical matters is uncertain, to say the least. To train a son or daughter for such a profession at the present time would, I consider, be a very poor investment for a parent looking for an adequate return upon his initial outlay.—I am, Sir, yours faithfully,

Gerald H. Fitzgerald.

Guy's Hospital, S.E. 1.

